# Experimental evolution to increase the efficacy of the entomopathogenic fungus *Beauveria bassiana* against malaria mosquitoes: Effects on mycelial growth and virulence

**DOI:** 10.1111/eva.12451

**Published:** 2017-04-14

**Authors:** Claudio A. Valero‐Jiménez, Jan A. L. van Kan, Constantianus J. M. Koenraadt, Bas J. Zwaan, Sijmen E. Schoustra

**Affiliations:** ^1^ Laboratory of GeneticsWageningen UniversityWageningenThe Netherlands; ^2^ Laboratory of EntomologyWageningen UniversityWageningenThe Netherlands; ^3^ Laboratory of PhytopathologyWageningen UniversityWageningenThe Netherlands

**Keywords:** *Beauveria bassiana*, entomopathogenic fungi, experimental evolution, malaria mosquitoes, mycelial growth rate, virulence

## Abstract

Entomopathogenic fungi such as *Beauveria bassiana* are currently considered as a potential control agent for malaria mosquitoes. The success of such strategies depends among others on the efficacy of the fungus to kill its hosts. As *B. bassiana* can use various resources for growth and reproduction, increasing the dependency on mosquitoes as a nutritional source may be instrumental for reaching this goal. Passage of entomopathogenic fungi through an insect host has been shown to increase its virulence. We evaluated the virulence, fungal outgrowth, mycelial growth rate, and sporulation rate of two *B. bassiana* isolates (Bb1520 and Bb8028) that underwent 10 consecutive selection cycles through malaria mosquitoes (*Anopheles coluzzii*) using an experimental evolution approach. This cycling resulted in an altered capacity of evolved *B. Bassiana* lineages to grow on different substrates while maintaining the ability to kill insects. Notably, however, there were no significant changes in virulence or speed of outgrowth when comparing the evolved lineages against their unevolved ancestors. These results suggest that fungal growth and sporulation evolved through successive and exclusive use of an insect host as a nutritional resource. We discuss the results in light of biocontrol and provide suggestions to increase fungal virulence.

## 1 Introduction

*Beauveria bassiana* is a cosmopolitan ascomycete fungus which is able to live as a saprophyte in the soil, as an endophyte in plants, and as an entomopathogen affecting a broad range of arthropods (Rehner et al., 2011). It is used as a biocontrol agent for different arthropods that are a pest in agriculture such as whiteflies, aphids, beetles, and locusts (Li et al., 2010). The use of *B. bassiana* is currently investigated for the potential control of malaria mosquitoes (Kanzok & Jacobs‐Lorena, 2006; Knols, Bukhari, & Farenhorst, 2010; Thomas & Read, 2007). There is a pressing need to develop novel tools to control malaria mosquitoes (*Anopheles* spp.), due to the rapid development of insecticide resistance in mosquito populations (Ranson & Lissenden, 2016). The natural occurrence of *B. bassiana* on mosquitoes has been seldom reported (Clark et al., 1968; Jenkins, 1964); nevertheless, *B. bassiana* could be a suitable alternative tool because extensive research has proven that it is effective in reducing malaria transmission under laboratory and field conditions by reducing the population size of the vector (Blanford et al., 2005; Mnyone et al., 2012; Scholte et al., 2005), including insecticide‐resistant mosquitoes (Farenhorst et al., 2009; Howard, Koenraadt, Farenhorst, Knols, & Takken, 2010; Howard et al., 2011). Furthermore, fungal biopesticides induce a late‐acting mortality, in which adult mosquitoes die at about 4–10 days after fungal infection. This is before the malaria parasite can be transmitted (Thomas & Read, 2007), but after reproduction of the mosquitoes, thereby reducing the effectiveness of selection for resistance of mosquitoes to fungal infection as this selection pressure only starts toward the end of life (in a so‐called selection shadow). Fungal infection also causes sublethal effects on the mosquitoes including reduced host‐seeking behavior and feeding propensity (Blanford et al., 2011; Scholte, Knols, & Takken, 2006), which potentially further reduces malaria parasite transmission.

Currently, one of the major hurdles that affect the usage of entomopathogenic fungi in field applications is their relatively poor and unpredictable performance compared to traditional insecticides. This is partly caused by variations in environmental factors such as temperature, humidity, and UV exposure that affect the fungal viability, as well as persistence, and virulence (Jaronski, 2010). Particularly, while chemical insecticides have a long residual effect, fungal biological control agents need to be applied more frequently which requires more efficient and complicated logistics, and mass production of biological material. To overcome some of these issues, genetic modification has been implemented to increase the ability to kill insects and tolerate adverse environmental conditions. For example, overexpression of the chitinase gene *Bbchit1* of *B. bassiana* resulted in increased infection efficiency and accelerated fungal infection (Fang et al., 2005). Expression of a gene encoding the insect‐specific scorpion neurotoxin AAIT in a *B. bassiana* isolate was shown to enhance virulence compared to the wild‐type isolate (Lu, Pava‐Ripoll, Li, & Wang, 2008). Fungal tolerance to oxidative stress has been achieved by overexpression of an endogenous superoxide dismutase that is involved in the detoxification of reactive oxygen species (Xie, Wang, Huang, Ying, & Feng, 2010). Other approaches have focused on targeting host‐specific molecules to interfere with normal insect development, for example, using an isolate expressing a trypsin‐modulating oostatic factor which showed increased virulence against *Anopheles gambiae* (Kamareddine, Fan, Osta, & Keyhani, 2013).

Although genetic modification for enhancement of virulence has shown encouraging results, field application of genetically modified organisms is associated with regulatory and societal issues that currently limit its usefulness (Koenraadt & Takken, 2011). Therefore, exploiting technical and biological (non‐GMO) resources is a feasible and a realistic alternative to increase the efficacy of fungal biological control agents. In this regard, various efforts have been made to improve the delivery of spores to the insect, including UV protectants and humidity stabilizers, and using different growth substrates that increase sporulation, virulence, and stress resistance (Jaronski, 2010). Increased tolerance to high temperature has been achieved for the fungus *Metarhizium anisopliae* through directed evolution using continuous culture (de Crecy, Jaronski, Lyons, Lyons, & Keyhani, 2009). Viaud, Couteaudier, and Riba (1998) created somatic hybrids from *B bassiana* and *Beauveria sulfurescens* that were more virulent and stable after cycling through an insect. Using artificial selection, a commercial isolate of *B. bassiana* became resistant to fungicides without reducing its virulence (Shapiro‐Ilan, Reilly, Hotchkiss, & Wood, 2002). The use of UV irradiation has also been used to obtain mutants that were 2‐deoxy‐d‐glucose resistant and that showed increased virulence (Robledo‐Monterrubio, Alatorre‐Rosas, Viniegra‐González, & Loera, 2009).

To date, there are no reports on the exploitation of spontaneous genetic changes combined with natural selection to increase the efficiency of *B*. *bassiana* as a biological control agent (except Quesada‐Moraga & Vey, 2003). When performed under controlled conditions in the laboratory, this approach is called experimental evolution, and it can in principle be used to alter fungal traits through spontaneous mutation and natural selection, and it can also be used to study adaptation, estimate evolutionary parameters, and test diverse evolutionary hypotheses (Kawecki et al., 2012; Schoustra, Punzalan, Dali, Rundle, & Kassen, 2012). Increased virulence is a usual outcome of experimental evolution studies aimed at adapting parasites to a new host (Ebert et al., 1998). Furthermore, the use of experimental evolution is potentially an effective tool to identify genes and mechanisms underpinning natural variation in virulence (Valero‐Jiménez, Wiegers, Zwaan, Koenraadt, & van Kan, 2016).

**Figure 1:**
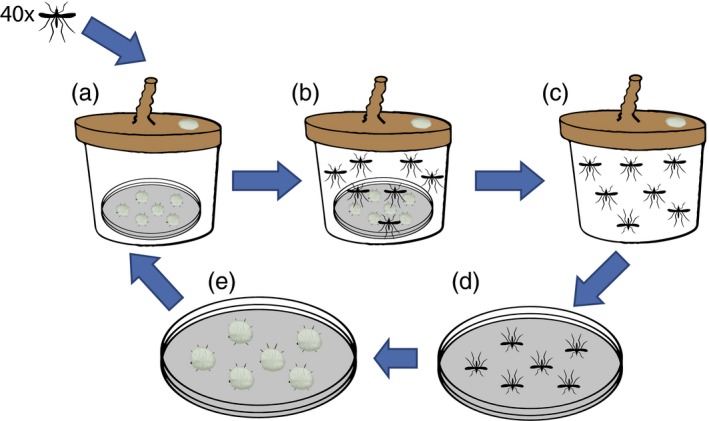
Setup of the experimental evolution assays that were used to evolve different *Beauveria bassiana* isolates using *Anopheles coluzzii* as the sole nutritional resource for 10 consecutive selection cycles. For every selection cycle (a), 40 uninfected *Ano. coluzzii* were exposed to twelve sporulating conspecific mosquitoes (mosquitoes that died of a fungal infection) in a bucket and then closed with a nylon sock. The mosquitoes were kept for 24 hr (b), to get a new infection. After this period, mosquitoes were transferred to a clean bucket (without sporulating mosquitoes) and mortality was recorded for 8 days (c). Mosquitoes that died after 4–6 days were transferred to a petri dish with moist filter paper and incubated at 27°C (d). After 8–9 days, dead mosquitoes were fully covered with fungal spores and were used as inoculum for the next cycle (e). There were five replicated evolving lineages per starting ancestor, and each replicate was considered as a separate evolutionary lineage. All buckets were kept in a climate‐controlled chamber (27 ± 1°C, 80% ± 10% RH, 12 hr L:D). Mosquitoes were fed ad libitum with a 6% glucose solution on a cotton plug provided on top of the nylon stocking

In this study, we evaluated (i) whether *B. bassiana* is suitable for experimental evolution by assaying the potential to evolutionary changes of growth parameters and (ii) whether it is possible to select for higher fungal virulence against mosquitoes. To this end, we used an experimental evolution approach in which *B. bassiana* was forced to use malaria mosquitoes as the sole nutritional resource for 10 consecutive selection cycles of around 80 fungal mitotic generations each (Figure 1). By comparing evolved lineages with their respective ancestors, we examined whether and how the phenotypic changes, which occurred using a natural selection regime, altered the virulence of *B. bassiana* toward mosquitoes. Specifically, we first test whether our selection protocol allows for phenotypic changes in evolving fungal lineages by measuring changes in characteristics such as mycelial growth rate (MGR) and sporulation rate (SR). Secondly, we test our evolved lineages for changes in fungal virulence against mosquitoes and discuss our results in light of biocontrol strategies.

## 2 Materials and Methods

### 2.1 Mosquito rearing and fungal isolates

*Anopheles coluzzii* mosquitoes used in the experiments originated from Suakoko, Liberia, and were reared in Wageningen University since 1989 under standard laboratory conditions. Adults were maintained in climate‐controlled rooms (27 ± 1°C, 80% ± 10% RH, and a 12‐hr L:D) and fed ad libitum on a 6% glucose solution. The fungal isolates used were *B. bassiana* ARSEF Bb1520 (isolated from *Lygus* sp. in France) and ARSEF Bb8028 (isolated from *Anthocoris nemorum* in Denmark), obtained from the USDA‐ARS Collection of Entomopathogenic Fungal Cultures (ARSEF). Initially, the fungus was grown on Sabouraud Dextrose Agar with 1% yeast extract (SDAY) for 14 days at 27°C, and spores were harvested with a 0.05% Tween‐80 solution to make a spore suspension. This suspension was kept at −80°C until use.

### 2.2 Initial bioassay

Two fungal isolates were subjected to experimental evolution and were chosen based on their natural difference in virulence, as established in a previous study (Valero‐Jiménez et al., 2014). Isolate Bb8028 is highly lethal to mosquitoes, while isolate Bb1520 is considered as having intermediate lethality. The initial bioassay was performed as described in Valero‐Jiménez et al. (2014). In brief, for each isolate, a fungal spore solution was applied to an A4‐sized paper one day before exposure and left to dry in a fume hood. Spore viability was checked on SDAY plates after 18–20 hr at 27°C, and spores with a detectable germ tube were considered viable. The coated papers were placed in PVC tubes (15 cm height and 8 cm diameter) that were sealed with cling film on both ends, and five replicate PVC tubes were used for each isolate tested. As a control, papers were coated with Shellsol T^®^ oil only. Forty *Ano. coluzzii* female mosquitoes (3–5 days old) were transferred to each PVC tube with the coated papers and exposed for 3 hr. Subsequently, mosquitoes were transferred to clean buckets (25 cm height and 20 cm diameter), which were sealed with a nylon sock. All buckets were kept in a climate‐controlled chamber (27 ± 1°C, 80% ± 10% RH, 12‐hr L:D). Mosquitoes were fed ad libitum with a 6% glucose solution on a cotton plug placed on top of the nylon stocking. Mortality was checked daily for 14 days, and fungal infection of dead mosquitoes was checked by dipping them for 5 s in 70% ethanol, incubating them on moist filter paper in sealed petri dishes at 27°C for 7–10 days, and inspecting them for visible fungal outgrowth.

### 2.3 Experimental evolution setup

The experimental evolution setup was based on the dead mosquitoes from the initial bioassay, in which fungal spores that grew out of dead mosquitoes (hereafter called sporulating mosquitoes) were used as an inoculum to infect a new batch of mosquitoes (Figure 1). A petri dish without a lid was placed at the bottom of a bucket, which was then closed with a nylon sock. Each petri dish contained twelve sporulating mosquitoes that died 4–6 days after exposure and that were subsequently incubated for 8–9 days. Forty *Ano. coluzzii* female mosquitoes (3–5 days old) were transferred to the bucket and exposed to sporulating mosquitoes for 24 hr. After this period, mosquitoes were transferred to a clean bucket (without sporulating mosquitoes), and mortality was recorded daily for 8 days. This recorded mortality was used to monitor evolutionary changes. There were five replicated evolving lineages per isolate, and each replicate was considered as a separate evolution lineage. As a nonevolving control, three replicate populations of mosquitoes were exposed to an empty petri dish to provide a baseline for mortality in each round of selection. All buckets were kept in a climate‐controlled chamber (27 ± 1°C, 80% ± 10% RH, 12‐hr L:D), and mosquitoes were fed ad libitum with a 6% glucose solution on a cotton plug. This experimental procedure lasted two weeks and was repeated for ten selection cycles in total. After each cycle, dead sporulating mosquitoes that had died 4–6 days after infection were put on SDAY plates for 14 days at 27°C. Spores were harvested with a 0.05% Tween‐80 solution to make a spore suspension and then kept at −80°C to have a fossil record of each cycle. This fossil record was used in a final bioassay that simultaneously tested the virulence of fungal spores from the 10th cycle (evolved) against the spores of the ancestors at the same time and using the same batch of mosquitoes. This was carried out to verify the results of changes in mortality rate due to fungal adaptation in the course of the various rounds of the evolutionary experiment, and to minimize the effect of variation in the rearing and experimental procedures during the time of the experiment. We conducted the final bioassay in a similar way as the initial bioassay, and we chose two evolutionary lineages for each ancestral isolate (lineages 2 and 5 originating from Bb1520 and lineages 3 and 4 originating from Bb8028).

### 2.4 Characterization of evolved fungal lineages

The MGR and the SR of all lineages derived from each ancestral isolate were measured and compared against the ancestor of each lineage. MGR and SR were chosen as they represent common measures for determining fitness in filamentous fungi (Pringle & Taylor, 2002; Schoustra, Debets, Slakhorst, & Hoekstra, 2006; Schoustra et al., 2012; Zhang et al., 2015). The MGR and SR were measured on four different media that differed in carbon source. The fungus was grown on Sabouraud Dextrose Agar with 1% yeast extract (SDAY) containing 40 g glucose, 10 g peptone, 10 g yeast extract, 15 g agar in 1 L of distilled water; trehalose medium containing 40 g trehalose, 10 g peptone, 10 g yeast extract, 15 g agar in 1 L of distilled water; insect medium containing minimal medium (0.4 g KH_2_PO_4_, 1.8 g Na_2_HPO_4_·2H_2_O, 0.6 g MgSO_4_·7H_2_O, 1 g KCL, 0.7 g NH_4_NO_3_) complemented with 20 g of crushed dead pupae of silkworms and 15 g agar in 1 L of distilled water; hexadecane medium containing minimal medium with *n*‐hexadecane (1 ml of a 20% solution diluted in hexane) as carbon source and 15 g agar in 1 L of distilled water. The media were chosen as representation of the conditions that the fungus was exposed to during the experimental evolution. SDAY was used as the reference medium for culturing of *B. bassiana*. Trehalose was chosen as it is the main disaccharide in the insect hemolymph. Whole dead insects were chosen because they simulate the complexity of nutrients found on mosquitoes, and hexadecane was selected because it is one of the main hydrocarbons present on the outer cuticle of insects. We hypothesized that lineages Bb1520 and Bb8028 that have undergone several rounds of experimental selection in mosquitoes will grow faster and/or produce more spores in the trehalose, hexadecane, and insect media than their unevolved ancestors. Growth and SR on SDAY will either be the same or decline in the evolved lineages (the latter as a result of potential trade‐offs between the use of contrasting nutritional sources). Spores were grown on SDAY petri dishes for 4 days, and later, agar plugs were used to inoculate the plates. Three plates were inoculated for each combination of lineage, evolutionary treatment (ancestor/evolved), isolate, and medium. After two weeks, MGR was determined by measuring the colony diameter (in mm) in two perpendicular directions (Schoustra et al., 2012). To determine SR, spores were harvested from a 3‐week‐old culture with 0.05% Tween‐80 solution and serially diluted on a 96‐well plate. Three dilutions were plated for each isolate/medium on SDAY plates and incubated for 72 hr at 27°C. Colonies were counted, and the cfu/ml was determined.

### 2.5 Quantification of fungal growth rate on dead mosquitoes

To determine whether there was a change in growth rate after a mosquito died between the evolved lineages and their ancestors, we conducted an experiment to quantify the amount of fungal genomes present at three time points, based on the method developed by Bell, Blanford, Jenkins, Thomas, and Read (2009). Lineage four of isolate Bb8028 from the 10th cycle and spores of Bb8028 from the initial bioassay were used. Mosquitoes were infected following a similar procedure as in the initial bioassay, in which three replicates of 40 mosquitoes were infected for each evolutionary treatment (ancestor/evolved). Infected mosquitoes, which died from days 5–7, were incubated at 27°C in petri dishes in groups of 9–12 mosquitoes per replicate and evolutionary treatment. A subset of 28 mosquitoes were removed per replicate and evolutionary treatment from the petri dishes after two, four, or six days of incubation and frozen at −80°C. DNA isolation was performed pooling four mosquitoes using the standard protocol from DNeasy plant mini kit (Qiagen) and following the recommendations from Bell et al. (2009). Real‐time quantitative PCR (RT‐qPCR) was performed using specific primers for *B. bassiana* that amplify part of the second internal transcribed spacer (ITS2) region of the rRNA gene (forward: GCCGGCCCTGAAATGG; reverse: R: GATTCGAGGTCAACGTTCAGAAG). Absolute quantification of fungal load was determined by comparing *C*_t_ values against a standard curve, generated from dilutions of DNA aliquots from isolate Bb8028 starting from 10^8^ conidia. Three replicates of each DNA standard (10^3^–10^7^ conidia) were included in each RT‐qPCR run.

### 2.6 Statistical analysis

Mosquito survival in the initial bioassay was analyzed using Kaplan–Meier survival analysis in SPSS (v.22) with significant differences between different isolates estimated using a log rank test (Valero‐Jiménez et al., 2014). Mosquito survival in the selection experiment was conducted in R (3.2.0) using a fixed effects Cox proportional hazards model, in which evolutionary treatment was used as a fixed effect, and lineage as a random effect. To detect differences in MGR and SR, we fitted general linear mixed models, with the sporulation data being log‐transformed before analysis. Evolutionary treatment (ancestor/evolved) and isolate (Bb1520/Bb8028) were used as fixed effects, whereas lineage (1–5) was incorporated as random effects to account for nonindependence. We adjusted the model to include interactions between all fixed effects and performed model comparisons to find the minimal adequate model. Differences between ancestor and evolved lineages were tested for significance using a Bonferroni correction, taking into account the multiple comparisons. To detect differences between the numbers of fungal genomes of infected mosquitoes, we fitted a general linear mixed model, in which isolate and time of sampling (day 2, day 4, or day 6) were used as fixed effects, and replicate (lineage) was incorporated as a random effect.

## 3 Results

### 3.1 Initial bioassay

**Figure 2:**
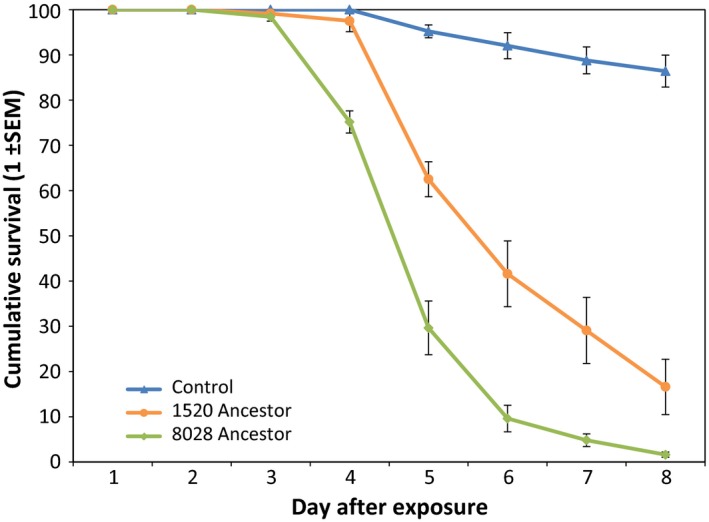
Daily proportional survival of *Anopheles coluzzii* infected with spores of different *Beauveria bassiana* isolates. In the control treatments, mosquitoes were exposed to only Shellsol T^®^ oil. Isolates Bb1520 and Bb8028 were used in the initial bioassay as a starting point for the experimental evolution. Data show means ± *SEM* from three replicates of 40 female mosquitoes

Fungal isolates Bb1520 and Bb8028 were used to set up the evolution experiment of 10 successive selection cycles through female *Ano. coluzzii* as a host with five replicated evolving lineages per starting isolate. The extent of virulence of each ancestral isolate was corroborated in an initial bioassay (Figure 2), and the spores that grew out of the dead mosquitoes served as starting material for experimental evolution. The mosquito survival differed depending on the isolate used (χ^2^ = 48.73, *p* < .001), in which isolate Bb8028 (high lethality) was significantly more virulent than isolate Bb1520 (intermediate lethality) in agreement with our previously published results (Valero‐Jiménez et al., 2014).

### 3.2 Characterization of evolved fungal lineages

**Figure 3:**
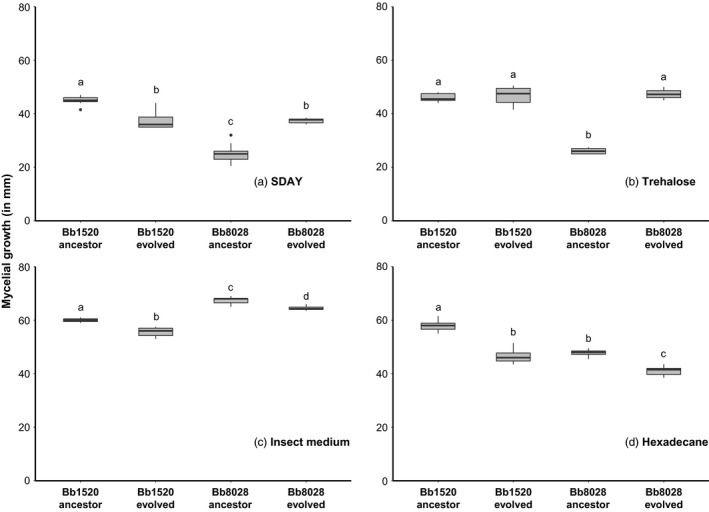
Box plot graphs representing the mycelial growth rate of five replicates of *Beauveria bassiana* grown on four different media that differ in the carbon source: SDAY (a), trehalose (b), insect medium (c), and hexadecane (d). The box plots shows the median (black line), the box boundaries mark the 25th and 75th quartiles, and the whiskers show the maximum and minimum values, while the dot represents the outliers. Treatments with the same lowercase letter are not significantly different in post hoc comparisons (Tukey's HSD,* p *=* *.05)

The ancestral and evolved lineages of isolates Bb1520 and Bb8028 were grown on four media that mainly differed in the carbon source, and served as conditions representing the selection environment (the insect cuticle, the insect hemolymph, the dead mosquitoes). The MGR and SR were measured to test whether phenotypic changes had occurred during this period of experimental evolution (likely reflecting genetic changes, Lang et al., 2013; Fisher & Lang, 2016; Schoustra, Hwang, Krug, & De Visser, 2016). There were significant effects of isolate and evolutionary treatment (ancestor/evolved) on MGR, but the effects differed depending on the media used. In SDAY and trehalose media, a significant interaction was found between the evolutionary treatment and the isolate used (SDAY: *F*_1,8_ **= 50.97, *p* < .0001; trehalose: *F*_1,8_ **= 39.85, *p* < .0001). In the SDAY medium (Figure 3a), evolved lineages of Bb1520 (intermediate lethality) and Bb8028 (high lethality) significantly differed in MGR compared to their respective ancestors after using a Bonferroni correction (*p* = .016, <.001, respectively), but in opposite directions. The evolved Bb1520 had a significantly lower MGR, while the evolved Bb8028 had a considerably higher MGR than its ancestor. In the trehalose medium (Figure 3b), the evolved Bb8028 had a significantly higher MGR than its ancestor (*p *<* *.0001), while no significant differences were found between the evolved Bb1520 and its ancestor (*p *=* *.989). Furthermore, when the fungus was grown on the insect medium (Figure 3c), a significant effect was found on the evolutionary treatment used (*F*_1,10_ **= 16.21, *p *=* *.0024) both evolved isolates had a significantly lower MGR than their respective ancestors (*p *=* *.038, .019, respectively). When grown on hexadecane as a carbon source (Figure 3d), a significant effect was found on the evolutionary treatment used (*F*_1,9_ **= 38.23, *p *=* *.0002), in which both evolved isolates performed worse than their ancestor with regard to MGR (Bb1520: *p *<* *.001; Bb8028: *p *=* *.011).

**Figure 4:**
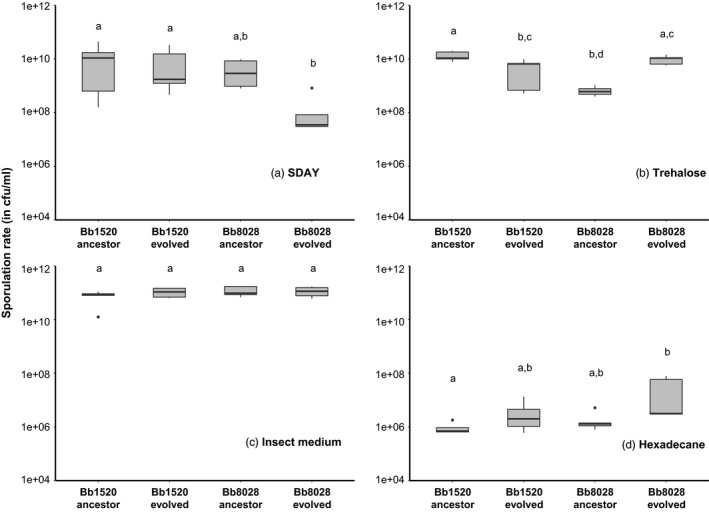
Box plot graphs representing the sporulation rate of five replicates of *Beauveria bassiana* grown on four different media that differ in the carbon source: SDAY (a), trehalose (b), insect medium (c), and hexadecane (d). The box plots show the median (black line), the box boundaries mark the 25th and 75th quartiles, and the whiskers show the maximum and minimum values, while the dot represents the outliers. Treatments with the same lowercase letter are not significantly different in post hoc comparisons (Tukey's HSD,* p *=* *.05)

The SR varied significantly in SDAY medium, and a significant interaction was found between the evolutionary treatment and isolate used (*F*_1,8_ = 4.876, *p *=* *.0267). The evolved lineages of Bb1520 and Bb8028 significantly differed in MGR compared to their respective ancestors (*p *<* *.001, .001, respectively). For trehalose medium, there is no overall effect of either evolutionary treatment or isolate used, but there is a crossover interaction (*F*_1,8_ = 10.36, *p *=* *.0123). The variation in SR when grown on insect medium and hexadecane (Figure 4c, d) was nonsignificant for both media (*p *=* *.358, .158, respectively). To further investigate the differences observed between ancestral and evolved isolates of Bb1520 and Bb8028, and to seek confirmation of our findings that evolution has occurred during our 10 selection cycles, fungal growth was measured on 35 different media differing in carbon source (Data S1). These media were selected from the Fungal Growth Database, which compares the growth of fungi on different carbon sources (de Vries, 2016). The MGR of the evolved Bb1520 lineage (Fig. S1a) was lower in 26 of the 35 media when compared to its ancestor, and for the remaining nine carbon sources, no discernible difference was observed. In contrast, the evolved lineage of Bb8028 (Fig. S1b) had a faster growth in 11 different media, eight with a lower growth, and 16 without a discernible difference in growth, when compared to its ancestor.

### 3.3 Mortality rate

**Figure 5:**
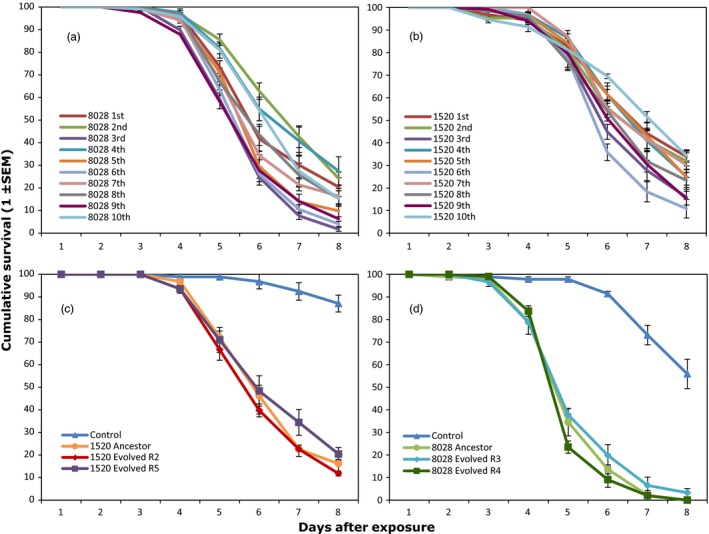
Daily proportional survival of *Anopheles coluzzii* infected with spores of different *Beauveria bassiana* isolates. In the control treatments, mosquitoes were exposed to only Shellsol T^®^ oil. During the course of the experimental evolution experiment, 10 selection cycles were completed and the daily mortality of mosquitoes was recorded for each cycle (a, b). Two lineages of the evolved isolates from Bb1520 (c) and Bb8028 (d) were tested against their respective ancestor. Data show means ± *SEM* from five (a, b) or three (c, d) replicates of 40 female mosquitoes

During the course of the evolution experiment, mosquito survival was measured during each selection cycle, and for both isolates, the median survival time varied along the 10 cycles of the experiment. Mosquitoes infected with isolate Bb8028 (Figure 5a) and isolate Bb1520 (Figure 5b) had a median survival time from 5 to 7 days, and 6 to 8 days, respectively. Infection rates (fungal infection in the form of sporulation after 5–7 days) were constant during the course of the evolution experiment, with an infection rate of 94.8%–99.4% and 98.1%–100% for Bb1520 and Bb8028, respectively. All the experimental evolution replicates for isolate Bb1520 (intermediate lethality) resulted in a similar mortality rate for the mosquitoes across the 10 selection cycles, except replicate 5 (hazard ratio, HR* *= 1.27 [±0.08], *p *=* *.003). In addition, all the experimental evolution replicates for isolate Bb8028 (high lethality) were similar, except replicate 4 (HR* *= 1.37 [±0.07], *p *<* *.0001), which had a significantly higher virulence across the 10 selection cycles. To further test whether there was a change in the virulence of the fungal isolates throughout the experimental evolution, we carried out a further experiment using two evolved lineages per starting ancestor (lineages 2 and 5 originating from Bb1520 and lineages 3 and 4 originating from Bb8028). These lineages were randomly chosen except lineage 5 from Bb isolate Bb1520 and lineage 4 from isolate Bb8028, which were handpicked because it was the only one whose evolved mortality was significantly higher than the ancestor one in the previous experiment. Replicates 2 and 5 from isolate Bb1520 (Figure 5c) were not significantly different from their ancestor in terms of their mortality to mosquitoes (HR* *= 1.14 [±0.16], *p *=* *.4; HR* *= 0.87 [±0.16], *p *=* *.4, respectively). Also, replicates 3 and 4 of isolate Bb8028 (Figure 5d) were not significantly different from their ancestor (HR* *= 0.82 [±0.15], *p *=* *.18; HR* *= 1.14 [±0.14], *p *=* *.35, respectively). Thus, while lineage 4 originating from Bb8028 seemingly had higher virulence when using measurements obtained during the selection experiment, the more direct assay comparing the virulence of the end point of our selection experiment with the ancestor did not confirm this result.

### 3.4 Quantification of fungal growth rate on dead mosquitoes

Evolutionary lineage 4 from isolate Bb8028 was used again in a bioassay, and the mortality rate was not significantly different from its ancestor (χ^2^ **= 1.84, *df *= 1, *p *=* *.175). Dead mosquitoes from this experiment were sampled two, four, and six days after their time of death. The fungal biomass (as measured as the number of copies of genomes) was not significantly different between the evolved and ancestor of isolate Bb8028 (*p *=* *.823). Nevertheless, the fungal biomass differed depending on the time sampled (*F*_1,11_ **= 37.56, *p *<* *.0001), in which there was a lower quantity of fungal biomass at day 2 compared to day 4 and 6 (Fig. S2).

## 4 Discussion

Increased efficacy of entomopathogenic fungi is an important prerequisite for their successful deployment as a biological control agent of insects. To this end, various approaches have been employed for investigating the underlying genetic and physiological mechanisms of fungal pathogenicity toward insects (reviewed in Valero‐Jiménez, Wiegers et al., 2016). In this study, we applied an experimental evolution approach in which *B. bassiana* was used to infect malaria mosquitoes for 10 consecutive selection cycles. We first assessed whether our selection scheme could result in the fixation of (general) phenotypic changes and, secondly, whether we could select for higher fungal virulence using this approach. While we did not perform any formal genetic analysis through crosses or sequencing to show that our observed phenotypic changes reflect genetic changes, we presume that phenotypic differences between haploid fungal strains assayed under the same conditions reflect genetic changes. This causal relationship has been shown for other evolutionary studies with fungi (and many other microbes; Fisher & Lang, 2016; Lang et al., 2013; Schoustra et al., 2016). We observed different MGR s and SRs between evolved and ancestral isolates when growing the fungus on different growth media, confirming that evolutionary changes had occurred during our experimental evolution. The direction of change in growth rate and SR in the evolved lineages originating from Bb8028 (high lethality) depended on the growth media, each of which reflect various (combinations of) components for fungal growth found in the insect. On the contrary, the evolved lineages originating from Bb1520 (intermediate lethality) on average had no change or a decrease in growth rate and sporulation. Similarly, Scully and Bidochka (2005) found that after five cycles through a host, there was a decreased ability of *Aspergillus flavus* to grow on an artificial nutrient source, suggesting that this could precede an adaptation to increase virulence on a given host.

In this study, we did not observe any significant change in virulence in 10 lineages originating from two isolates of *B. bassiana* after 10 selection cycles using malaria mosquitoes as a host. Previous studies reported mixed results. Some show that entomopathogenic fungi that have been serially passed through an insect host have acquired an increased virulence (Adames, Fernández‐ruvalcaba, Peña‐chora, & Hernández‐Velázquez, 2011; Hayden, Bidochka, & Khachatourians, 1992; Quesada‐Moraga & Vey, 2003; Song & Feng, 2011). For example, an isolate of *B. bassiana* increased in virulence after two cycles through *Locusta migratoria* (Quesada‐Moraga & Vey, 2003). However, similar studies have reported a different outcome that no enhancement of virulence of entomopathogenic fungi was observed after cycling through an insect hosts (Latch, 1976; Scully & Bidochka, 2005; Vandenberg & Cantone, 2004). For instance, three strains of *Paecilomyces fumosoroseus* after 15 cycles through insects did not acquire an increased virulence, and, in some cases, virulence was even reduced (Vandenberg & Cantone, 2004). These mixed results indicate that it is important to have a sufficient number of evolution cycles and that assessing phenotypic change includes comparing evolved lineages and the ancestor in one experimental assay. Moreover, (subtle) variation in the species used and the experimental protocol may result in shifts in which traits and aspects of the biological interaction between fungus and insect are actually under selection.

For our experimental evolution setup, we hypothesized that during selection cycles, beneficial mutations might arise that increase virulence and therefore be selectively enriched in the fungal population, especially as *B. bassiana* displays extensive natural variation in virulence against mosquitoes (Valero‐Jiménez et al., 2014). Nevertheless, when starting an evolution experiment with a clonal population, there is a delay in the detection of fitness changes in the population due to the low‐frequency emergence of beneficial mutations, random genetic drift, the magnitude of the selective advantage, and clonal interference (Long, Liti, Luptak, & Tenaillon, 2015). We also considered that during this experimental setup, not only an increase in virulence would be advantageous to *B. bassiana*, but also an increased rate in sporulation once the host is dead. As spores from a dead mosquito were used to inoculate a new batch of mosquitoes, a mutation that would increase sporulation would be selected over time. Nevertheless, we could not detect any differences in rate of fungal outgrowth after the host had died in lineage 4 of isolate Bb8028. This remains to be investigated for the other lineages of Bb1520 and Bb8028.

It is also important to consider that the theory for the evolution of virulence in pathogens is based on the assumption of a trade‐off between virulence and transmission. A parasite that reproduces at a low rate will not be harmful to the host but may allow for transmission of the parasite to other hosts, whereas a high level of parasite reproduction will be very harmful for the host but could impede transmission if this kills the host (too) quickly. The optimum would be an intermediate virulence level that maximizes transmission (Anderson & May, 1982; Ebert & Bull, 2003; Ewald, 2003; Rafaluk, Jansen, Schulenburg, & Joop, 2015). Under this model, we could hypothesize that in *B. bassiana,* a trade‐off exists between virulence and transmission, as high virulence (which could be due to high levels of toxin production, *cf*. Valero‐Jiménez, Faino et al., 2016) could lead to the death of mosquitoes before the fungus has colonized the entire host body and accumulated sufficient biomass to ensure maximal spore production (Boomsma, Jensen, Meyling, & Eilenberg, 2014). Therefore, one could speculate that the lack of observed increase in virulence in our experiment, even when using two different ancestral stains with different starting virulence, could mean that for our host–parasite (mosquito–fungus) combination, there is equilibrium between virulence and transmission and that the evolving fungi are close to that equilibrium.

In conclusion, our results show that prolonged selection of *B. bassiana* on malaria mosquitoes as the sole medium for growth can lead to evolutionary changes in fungal growth characteristics. These include changes in mycelial growth and SR, but not in fungal virulence. Infection of insects by *B. bassiana* is a complex trait that involves several steps including attachment of conidia to a host cuticle, germination of conidia, breaching of the cuticle, secretion of toxins, evasion of the immune system, and sporulation from the dead insect body (Valero‐Jiménez, Wiegers et al., 2016). Due to the complexity of this trait and the intrinsic variation of the insect host, we suggest that adjustments in the experimental setup are required to understand the components of fungal virulence and to be able to more targeted select for increased virulence. This increase in virulence should match the requirement that the mosquitoes do not die of their fungal infection before they have had the opportunity to reproduce. The latter is essential for ensuring that application of this evolved fungal strain is evolution‐proof concept. Nutrition is a key factor in immune function of insects (Lee, Simpson, & Wilson, 2008); therefore, it is unlikely that the immune response to fungal infection is constant over time. This is related to the general “quality” of the mosquitoes from our stock, which is known to vary between batches. This would significantly reduce the selection efficacy because mosquitoes would succumb from their poor fitness rather than because of high virulence of the fungal genotype with which it is infected. Thus, we suggest either using an insect for which quality is better controlled (e.g., *Drosophila melanogaster*) and/or that cycling through a dead insect host would give a better opportunity to detect significant differences of the evolved lineages using experimental evolution. Also, the use of a low‐virulent isolate as a starting ancestor would provide an increased selective benefit of increased virulence, therefore increasing the odds of detecting phenotypic changes in less selection cycles. To further study evolutionary changes using experimental evolution in *B. bassiana*, additional experiments should consider the results obtained in this study. Despite our results, the use of experimental evolution to naturally adapt to a particular host could still be a good tool to study host–pathogen interactions and understand the genetic mechanisms involved in fungal virulence. The understanding of genetic mechanisms will improve our methods to use *B. bassiana* as a viable biological control agent against malaria mosquitoes.

## Data Archiving Statement

Data available from the Dryad Digital Repository: http://dx.doi.org/10.5061/dryad.24kj1.
