# Supplementary figures and images for: Experimental evolution to increase the efficacy of the entomopathogenic fungus Beauveria bassiana against malaria mosquitoes: Effects on mycelial growth and virulence
Source: Evol Appl. 2017 Apr 14;10(5):433–43. doi: 10.1111/eva.12451 (PMC5427670; doi:10.1111/eva.12451)

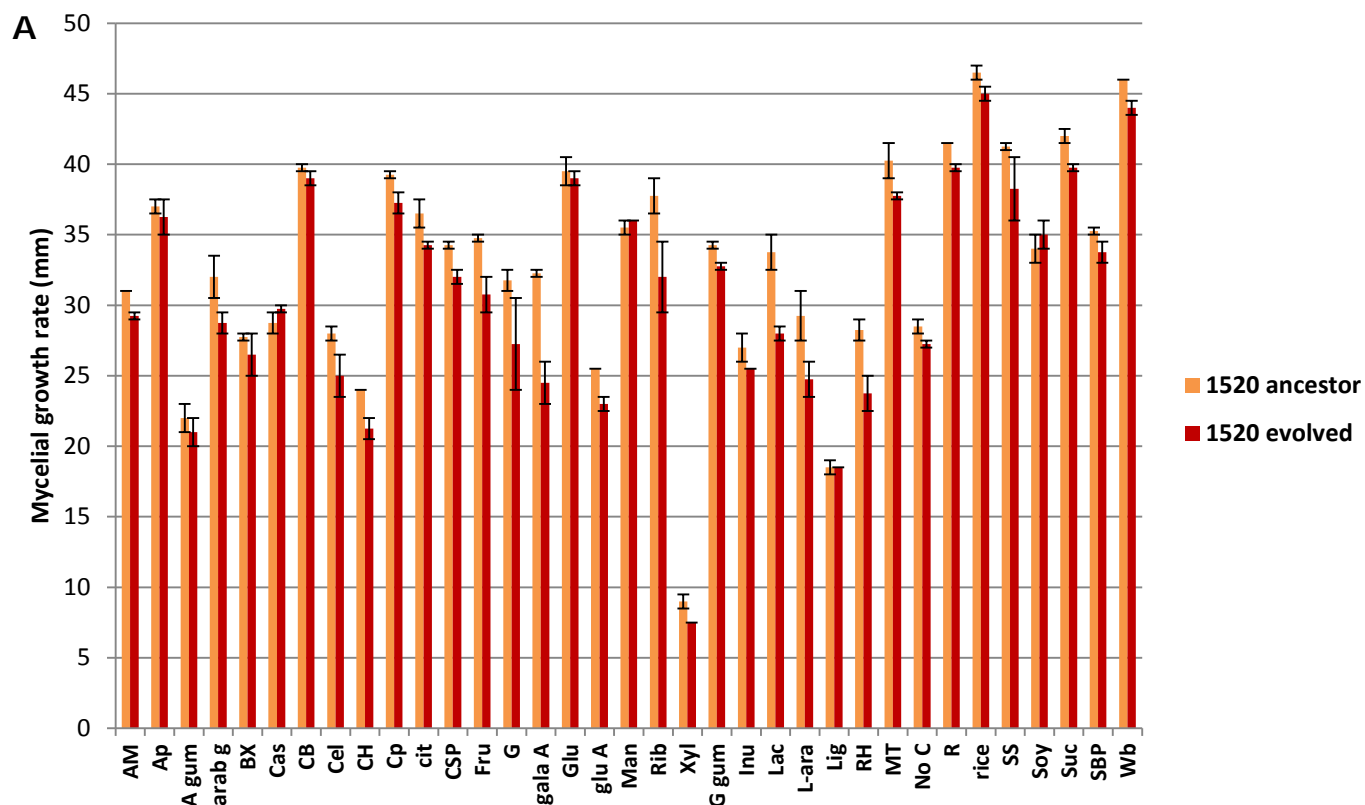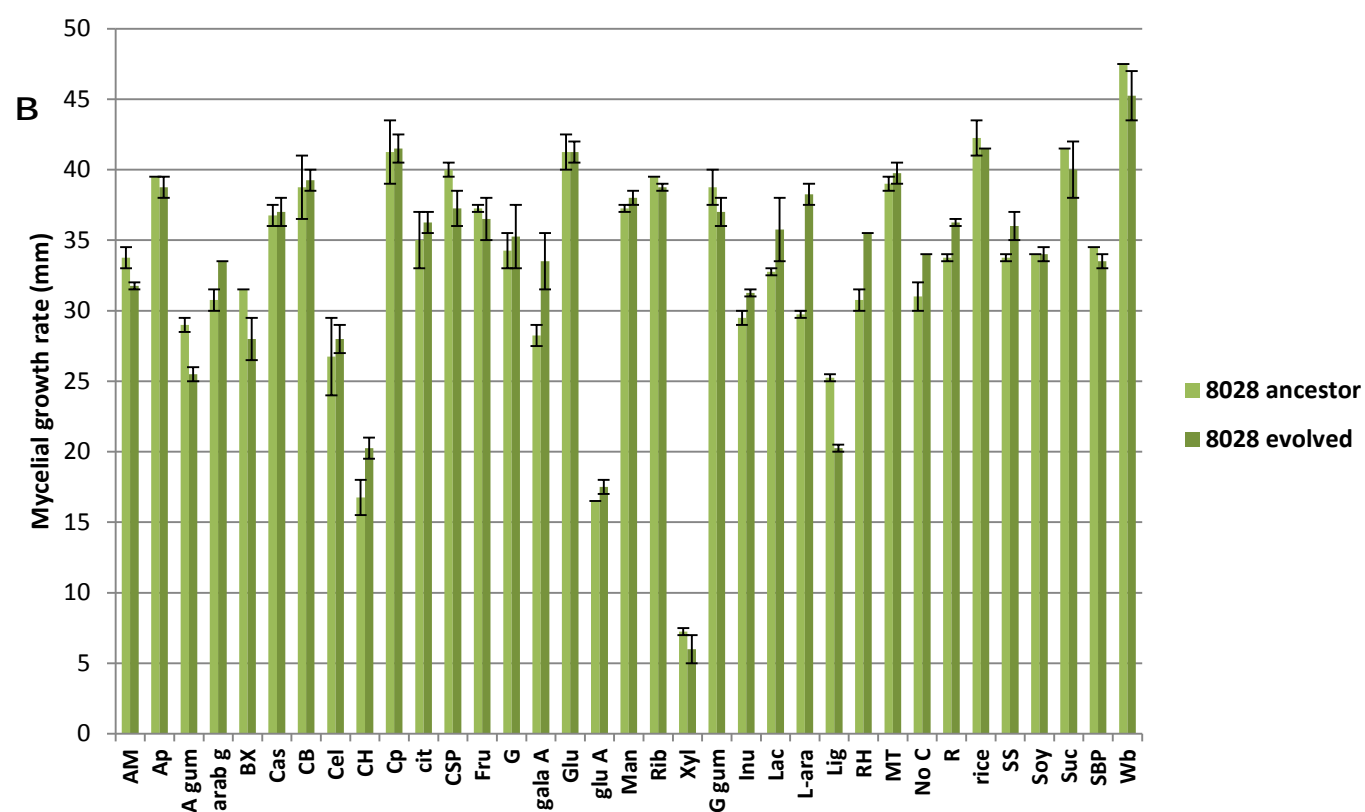

Supplement: Supplementary file 1 [file EVA-10-433-s001.pdf]

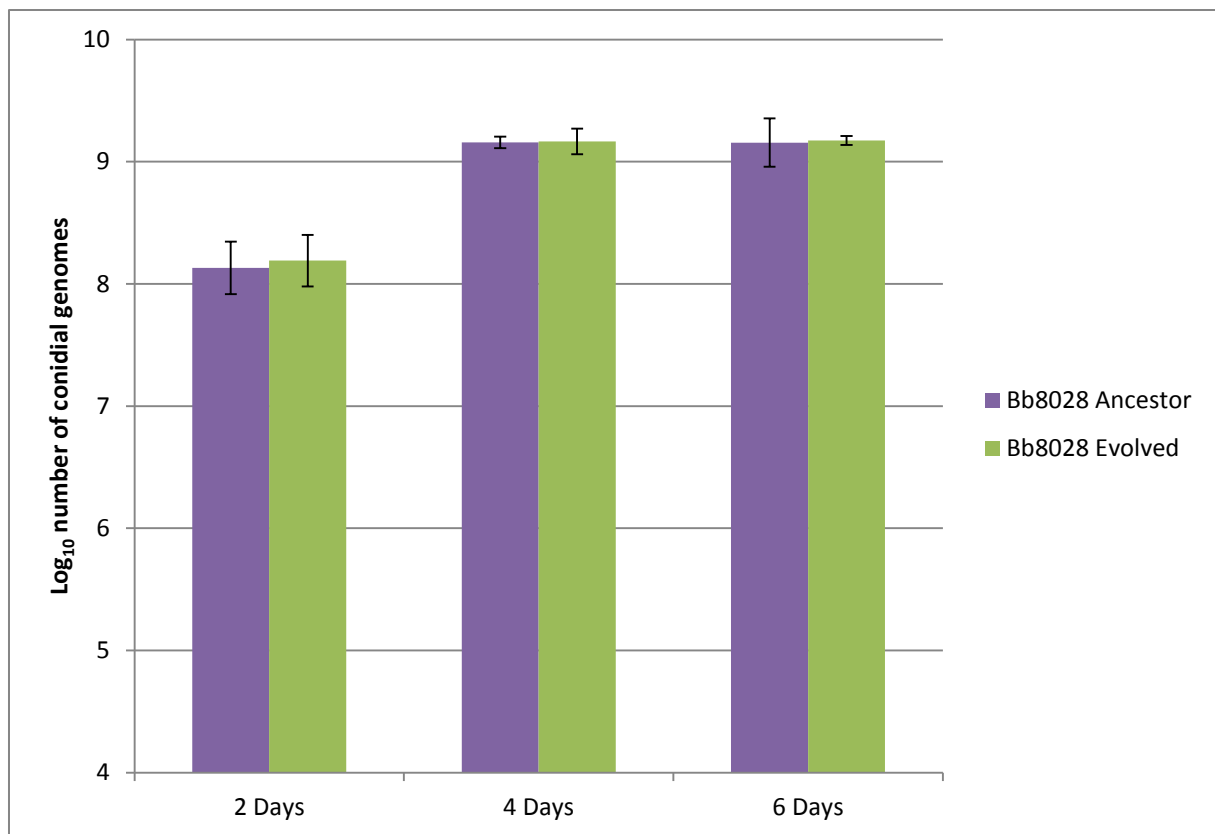

Supplement: Supplementary file 2 [file EVA-10-433-s002.pdf]
