# Supplementary material for: Experimental evolution to increase the efficacy of the entomopathogenic fungus Beauveria bassiana against malaria mosquitoes: Effects on mycelial growth and virulence
Source: Evol Appl. 2017 Apr 14;10(5):433–43. doi: 10.1111/eva.12451 (PMC5427670; doi:10.1111/eva.12451)
Supplement: Supplementary file 3 [file EVA-10-433-s003.docx]

**Supplementary materials**

**Data S1**

Fungal growth was measured when grown on 35 different media differing in the carbon source, in order to further investigate differences between ancestral and evolved lineages of Bb1520 and Bb8028. The fungi were grown on a minimal medium containing: 0.5 g MgSO_4_·7H_2_O, 1.5g L-aspargine (monohydrate), 1 ml of Solution A, 1 ml of Solution B, 1 ml of Solution C, 2.5 ml of Solution D, 5 ml of urea (1M), and select agar 15 g in 1 l of distilled water. Solution A consisted of thiaminiumdichloride (0.012 g/100 ml); Solution B: FeCl_3_·6H_2_O (0.5 g/100 ml); Solution C: HBO_3_ (0.06 g/l), (NH_4_)Mo_7_O_24_·4H_2_O (0.04 g/l), CuSO_4_·5H_2_O (0.2 g/l), ZnSO_4_·7H_2_O (2.0 g/l), MnSO_4_·4H_2_O (0.1 g/l), CoCl_2_·6H_2_O (0.4 g/l), and Ca(NO_3_)_2_·4H_2_O (1.2 g/l); Solution D: KH_2_PO_4_ (184 g/l), and K_2_HPO_4_·3H_2_O (524.05 g/l).

The minimum media was complemented with one source of carbon, that consisted of: 30 g alfalfa meal (AM), 10 g apple pectin (Ap), 10 g arabic gum (A gum), 10 g arabinogalactan (arab g), 10 g beechwood xylan (BX), 10 g casein (Cas), 8.5 g cellobiose (CB), 10 g cellulose (Cel), 10 g chitin (CH), 10 g citrus pectin (Cp), 30 g citrus pulp (cit), 30 g cotton seed pulp (CSP), 4.5 g D-fructose (Fru), 4.5 g D-galactose (G), 5.4 g D-galacturonic acid (gala A), 5 g D-glucose (Glu), 4.85 g D-glucuronic acid (glu A), 4.5 g D-mannose (Man), 3.75 g D-ribose (Rib), 3.75 g D-xylose (Xyl), 10 g guar gum (G gum), 10 g inulin (Inu), 9 g lactose (Lac), 3.75 g L-arabinose (L-ara), 10 g lignin hydrolytic (Lig), 4.5 g L-rhamnose (RH), 9 g maltose (MT), 14.85 g raffinose (R), 30 g rice bran (rice), 10 g soluble starch (SS), 30 g soybean hulls (Soy), 8.5 g sucrose (Suc), 30 g sugar beet pulp (SBP), 30 g and wheat bran (Wb).

**Figure S1.** Bar chart representing the mycelial growth rate of *Beauveria bassiana* grown on 35 different media that differ in the carbon source: alfalfa meal (AM), apple pectin (Ap), arabic gum (A gum), arabinogalactan (arab g), beechwood xylan (BX), casein (Cas), cellobiose (CB), cellulose (Cel), chitin (CH), citrus pectin (Cp), citrus pulp (cit), cotton seed pulp (CSP), D-fructose (Fru), D-galactose (G), D-galacturonic acid (gala A), D-glucose (Glu), D-glucuronic acid (glu A), D-mannose (Man), D-ribose (Rib), D-xylose (Xyl), guar gum (G gum), inulin (Inu), lactose (Lac), L-arabinose (L-ara), lignin hydrolytic (Lig), L-rhamnose (RH), maltose (MT), no carbon source (No C), raffinose (R), rice bran (rice), soluble starch (SS), soybean hulls (Soy), sucrose (Suc), sugar beet pulp (SBP), and wheat bran (Wb). Panel A shows the comparison of the ancestor and evolved lineage of Bb1520, while panel B shows the comparison of the ancestor and evolved lineage 4 of Bb8028. Data shown present the means ± SEM from two replicates.

**Figure S2.** Bar chart representing the number of *Beauveria bassiana* conidial units on/within infected mosquitoes with respect to time after death. Lineage four of isolate Bb8028 from the 10^th^ cycle and spores of Bb8028 from the initial bioassay were used. Data shown present the means ± SEM from three replicates.
